# CD4 count evolution of HIV-infected patients in follow-up as an indicator of quality of care

**DOI:** 10.7448/IAS.15.6.18102

**Published:** 2012-11-11

**Authors:** A Sasse, D Van Beckhoven, for the Belgian Research AIDS and HIV Consortium (BREACH)

**Affiliations:** Scientific Institute of Public Health, Brussels, Belgium

## Abstract

**Objective:**

To study the evolution of CD4 count of HIV-infected patients in follow-up as an indicator of quality of care.

**Methods:**

Adult patients newly diagnosed with HIV in 2007 who entered in care in the AIDS Reference Centres (ARC) and remained in care for at least one year were studied until end 2009. Optimal CD4 evolution was defined as having a CD4 count above 350 cells/mm^3^ after 1 year in HIV care, or an increasing rate exceeding 50 cells/mm^3^ per year, and this regardless of antiviral therapy. The proportion of patients with optimal CD4 evolution was measured and factors associated with outcome were identified by logistic regression.

**Results:**

482 patients were included. Median age was 37 years, 31.1% were females, 51.7% Belgians, 32.2% from Sub-Saharan Africa, 50.5% heterosexual, 48.7% MSM. 59.5% had a CD4 count above 350 at entry in care, 11.2% did not have a regular retention in care (at least 1 consultation/6-month period). 401 (83.2%) patients had an optimal CD4 evolution after 1 year in care. 60.5% of patients with non-optimal evolution had a CD4 count below 350 at entry in care. Although the proportion of female sex, heterosexual transmission, Sub-Saharan nationality and low retention in care was higher in the non-optimal CD4 evolution group compared to the optimal group, none of these characteristics showed a significant association with non-optimal CD4 count evolution.

**Conclusion:**

83.2% of patients had an optimal CD4 evolution after at least 1 year in HIV care. This indicator, analysed together with indicators of entry and retention in care, could contribute to a better monitoring of the HIV epidemic and to identify more precisely the steps in care system that could be improved. These indicators should be fully integrated in HIV surveillance.

**Table 1:** Characteristics associated with non optimal CD4 evolution among patients in care

	Optimal CD4 evolution	Non-optimal CD4 evolution	OR (95% CI)	Adjusted OR (95% CI)
**Gender (N, %)**				
Male	281 (70.1)	51 (63.0)	1	1
Female	120 (29.9)	30 (37.0)	1.38 (0.84–2.27)	0.89 (0.41–1.91)
**Age at diagnosis (N, %)**				
<40 yrs	252 (62.8)	51 (63.0)	1	1
≥40 yrs	149 (37.2)	30 (37.0)	0.99 (0.61–1.63)	1.06 (0.60–1.98)
**Way of transmission (N, %)**				
Heterosexual	162 (49.4)	37 (56.1)	1	1
MSM	163 (49.7)	29 (43.9)	0.78 (0.46–1.33)	0.85 (0.40–1.80)
IDU	3 (0.9)	0 (0.0)	/	
**Nationality (N, %)**				
Belgian	177 (52.8)	32 (46.4)	1	1
SSA	103 (30.8)	27 (39.1)	1.45 (0.82–2.56)	1.37 (0.62–3.01)
European	39 (11.6)	4 (5.8)	0.57 (0.19–1.70)	0.57 (0.19–1.73)
Other	16 (4.8)	6 (8.7)	2.07 (0.75–1.70)	1.74 (0.57–5.30)
**Retention in care (N, %)**				
≥1 consult./period	359 (89.5)	69 (85.2)	1	1
<1 consult./period	42 (10.5)	12 (14.8)	1.49 (0.74–2.97)	1.45 (0.68–3.12)

